# Supplementary material for: Osmostress enhances activating phosphorylation of Hog1 MAP kinase by mono‐phosphorylated Pbs2 MAP2K
Source: EMBO J. 2020 Feb 3;39(5):e103444. doi: 10.15252/embj.2019103444 (PMC7049814; doi:10.15252/embj.2019103444)
Supplement: Supplementary file 1 — Appendix [file EMBJ-39-e103444-s001.pdf]

Appendix

Osmostress enhances the activating phosphorylation of MAPK Hog1 by mono-phosphorylated MAP2K Pbs2

Kazuo Tatebayashi[1,2,3]*, Katsuyoshi Yamamoto[2], Taichiro Tomida[4], Akiko Nishimura[2,3], Tomomi Takayama[2,3], Masaaki Oyama[5], Hiroko Kozuka-Hata[5], Satomi Adachi-Akahane[4], Yuji Tokunaga[6], and Haruo Saito[2]*

[1]Laboratory of Molecular Genetics, Frontier Research Unit, Institute of Medical Science, The University of Tokyo, Tokyo 108-8639, Japan.

[2]Division of Molecular Cell Signaling, Institute of Medical Science, The University of Tokyo, Tokyo 108-8639, Japan.

[3]Department of Biological Sciences, Graduate School of Science, The University of Tokyo, Tokyo 113-0033, Japan.

[4]Department of Physiology, School of Medicine, Faculty of Medicine, Toho University, Tokyo 143-8540, Japan.

[5]Medical Proteomics Laboratory, Institute of Medical Science, The University of Tokyo, Tokyo 108-8639, Japan.

[6]Molecular Profiling Research Center for Drug Discovery, National Institute of Advanced Industrial Science and Technology, Tokyo 135-0064, Japan

NaCl

Molarity (M)	x_w	γ_w	a_w	Π (MPa)
0.2	0.9928	1.0008	0.9936	0.89
0.3	0.9892	1.0010	0.9902	1.35
0.4	0.9856	1.0013	0.9869	1.81
0.6	0.9785	1.0017	0.9802	2.76
0.8	0.9715	1.0019	0.9734	3.72
1.0	0.9645	1.0020	0.9664	4.70
1.2	0.9575	1.0019	0.9593	5.71
1.4	0.9506	1.0016	0.9521	6.75
1.6	0.9437	1.0011	0.9447	7.83
1.8	0.9369	1.0002	0.9371	8.94

Sorbitol

Molarity (M)	x_w	γ_w	a_w	Π (MPa)
0.4	0.9925	0.9998	0.9922	1.07
0.8	0.9842	0.9995	0.9838	2.25
1.2	0.9751	0.9990	0.9741	3.61
1.6	0.9654	0.9981	0.9636	5.11
2.0	0.9543	0.9966	0.9510	6.91
2.4	0.9422	0.9941	0.9367	9.01

Appendix Figure S1. Conversion of NaCl and sorbitol solute concentration (molarity, M) to osmotic pressure (MPa).

Osmotic pressure was calculated according to the following formulas.

$$\Pi = -(RT/V_w)\ln(a_w)$$

$$a_w = \gamma_w x_w$$

where Π is osmotic pressure (Pascal); R is the Gas constant (8.3145 J K^{-1} mol^{-1}); T is the absolute temperature (298.15 K at 25 °C); V_w is the molar volume of H_2O (18.01 cm^3); a_w is water activity of the solution (*i.e.*, chemical potential of the solvent water); γ_w is the activity coefficient of the solvent water; and x_w is the mole fraction of the solvent water. x_w, γ_w and/or a_w were obtained by interpolation of the data in the literature (Comesaña *et al.*, 2001, Fontana Jr, 2007, Koohyar *et al.*, 2013, Ninni *et al.*, 2000, Zhang & Han, 1996).

References

Comesaña JF, Correa A, Sereno AM (2001) Water activity in sorbitol or xylitol + water and sorbitol or xylitol + sodium chloride + water systems at 20°C and 35°C. *J Chem Eng Data* 46: 716-719

Fontana Jr AJ (2007) Watert activity of unsaturated salt solutions at 25°C. In Water activity in foods: Fundamentals and applications, Barbosa-Cánovas GV, Fontana Jr AJ, Schmidt SJ, Labuza TP (eds) pp 395-397.

Koohyar F, Rostami AA, Chaichi MJ, Kiani F (2013) Study on thermodynamic properties for binary systems of water + L-cysteine hydrochloride monohydrate, glycerol, and D-sorbitol at various temperatures. *J Chemistry* 2013: ID 601751

Ninni L, Camargo MS, Meirelles AJA (2000) Water activity in polyol systems. *J Chem Eng Data* 45: 654-660

Zhang HL, Han SJ (1996) Viscosity and density of water + sodium chloride + potassium chloride solutions at 298.15 K. *J Chem Eng Data* 41: 516-520
