# Supplementary material for: Osmostress enhances activating phosphorylation of Hog1 MAP kinase by mono‐phosphorylated Pbs2 MAP2K
Source: EMBO J. 2020 Feb 3;39(5):e103444. doi: 10.15252/embj.2019103444 (PMC7049814; doi:10.15252/embj.2019103444)
Supplement: Supplementary file 2 — Expanded View Figures PDF [file EMBJ-39-e103444-s002.pdf]

Expanded View Figures

A

NaCl

Molarity (M)	Π (MPa)
0.2	0.89
0.3	1.35
0.4	1.81
0.6	2.76
0.8	3.72
1.0	4.70
1.2	5.71
1.4	6.75
1.6	7.83
1.8	8.94

Sorbitol

Molarity (M)	Π (MPa)
0.4	1.07
0.8	2.25
1.2	3.61
1.6	5.11
2.0	6.91
2.4	9.01

B

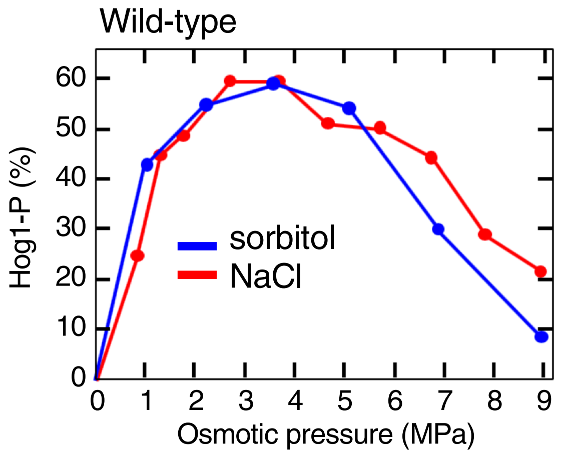

C

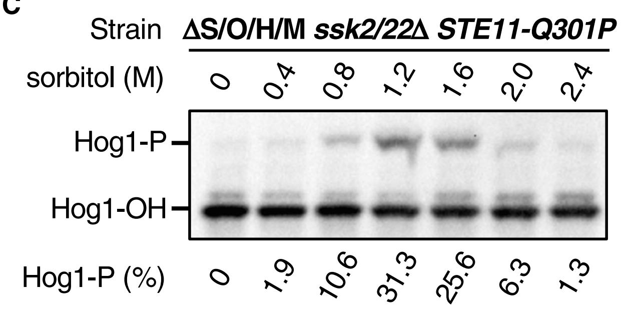

D

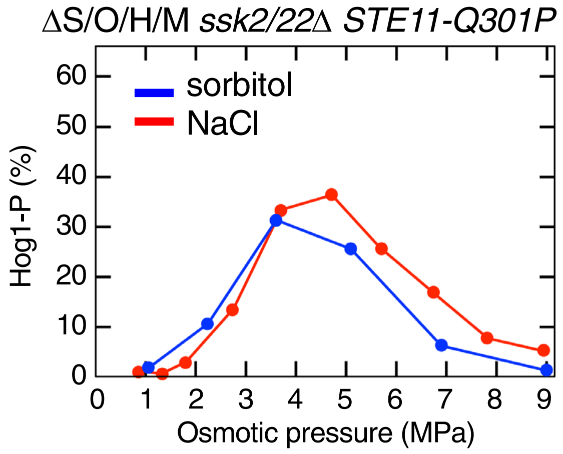

Figure EV1. The "downstream osmosensor" responds to osmostress regardless of the type of osmostressor.

A Conversion of NaCl and sorbitol solute concentration (molarity, M) to osmotic pressure Π (mega pascal, MPa). For more detail, see the Appendix Fig S1.

B Comparison of Hog1 phosphorylation induced by NaCl and sorbitol. The yeast strain TM142 (WT) was stimulated with various concentrations of NaCl or sorbitol for 5 min, and the percentage of Hog1-P was determined using a Phos-tag band-shift assay. Solution molar concentrations were converted to the corresponding osmotic pressures (MPa) using the table in (A).

C Phos-tag band-shift analyses of sorbitol-induced Hog1 phosphorylation. The yeast strain KY603-3 (ΔS/O/H/M *ssk2/22Δ STE11-Q301P*) was stimulated with the indicated concentrations of sorbitol for 5 min.

D Same as in (B), except that the yeast strain KY603-3 (ΔS/O/H/M *ssk2/22Δ STE11-Q301P*) was used.

Source data are available online for this figure.

 © 2020 The Authors

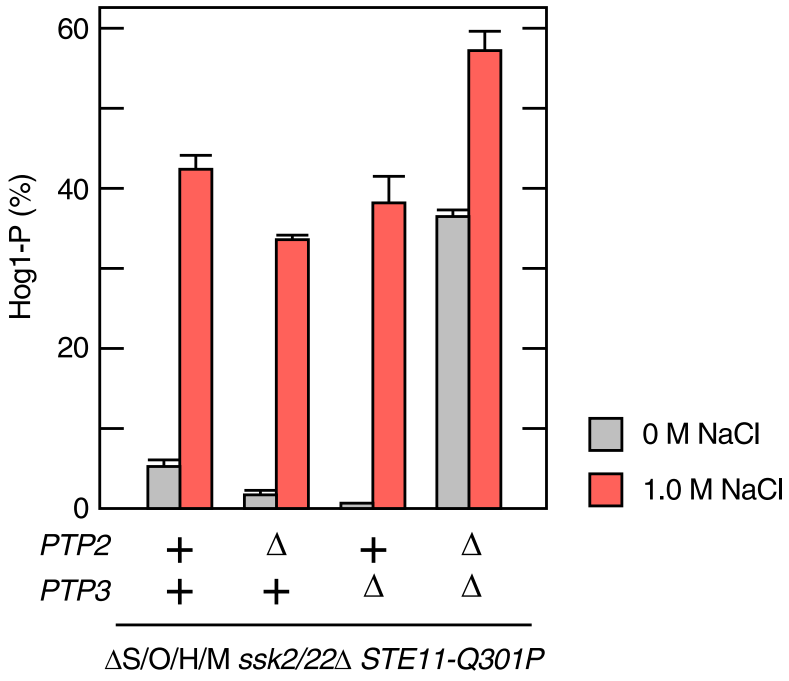

Figure EV2. Protein tyrosine phosphatases Ptp2/Ptp3 are not involved in the osmotic enhancement of the Pbs2-Hog1 reaction.

Yeast strains of the indicated genotypes (shown below the graph) were grown exponentially and were either untreated or exposed to 1.0 M NaCl for 5 min. Hog1 phosphorylation was determined using the Phos-tag band-shift assay. Strains used are KY603-3 (ΔS/O/H/M *ssk2/22Δ STE11-Q301P*) and its derivatives, KT303 (*ptp2Δ*), KT307 (*ptp3Δ*), and KT305 (*ptp2Δ ptp3Δ*). Data information: Error bars are SEM (n = 3).

© 2020 The Authors

A

```
             514 518
              |   |
Sc Pbs2   ..NLVASLAKTN-IGCQSYMAPER..
Sc Ste7   ..KLINSIADTF-VGTSTYMSPER..
Hs Mkk3   ..YLVDSVAKTMDAGCKPYMAPER..
Hs Mkk4   ..QLVDSIAKTRDAGCRPYMAPER..
Hs Mek1   ..QLIDSMANSF-VGTRSYMSPER..
```

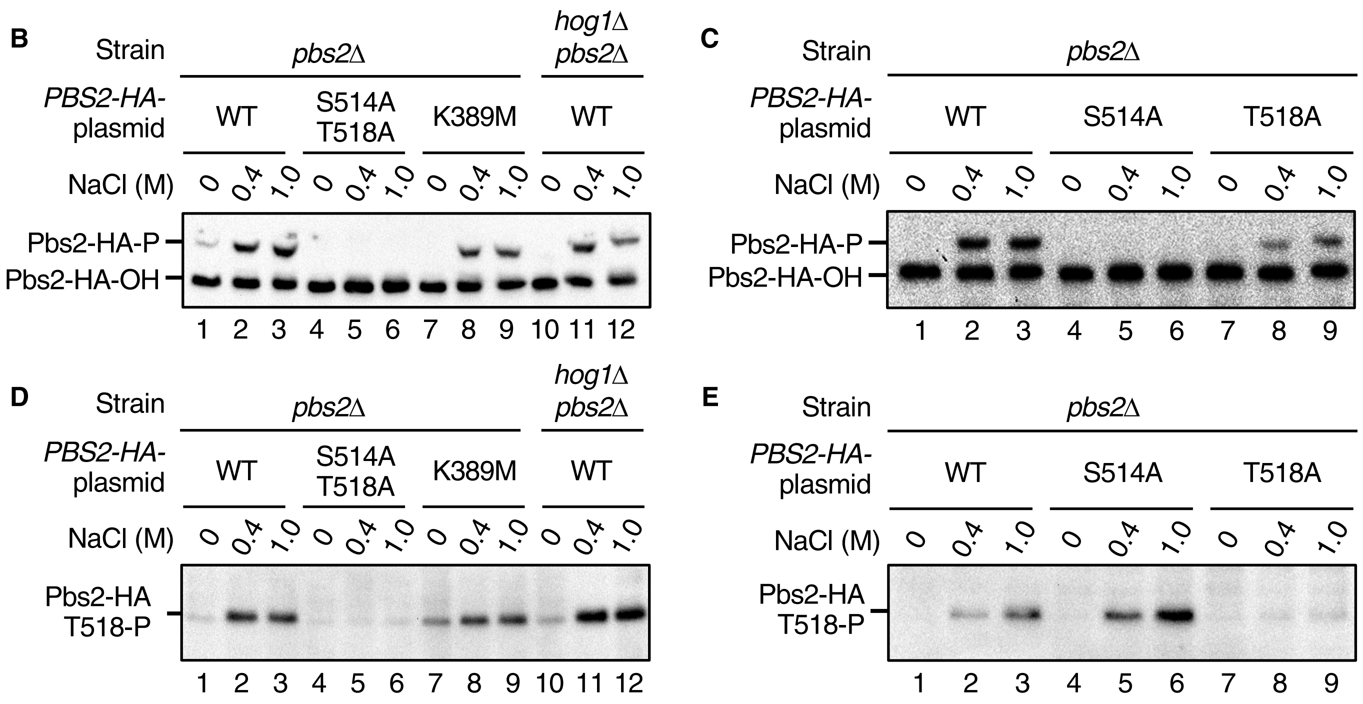

F

Peptides identified				
	strain	*ssk2/22Δ*	*ssk2/22Δ*	*ste11Δ*
	NaCl for 7 min	0 M	0.6 M	0.6 M
**S514**	LCDFGVSGNLVASLAK	6	15	7
	LCDFGVSGNLVApSLAK	0	0	5
**T518**	TNIGCQSYMAPER	3	3	4
	pTNIGCQSYMAPER	0	0	0

Figure EV3.

 © 2020 The Authors

Figure EV3. Detection of the activating phosphorylation at S514 and T518 in Pbs2.

A Alignment of the amino acid sequences around the activating phosphorylation sites in yeast (Sc) and human (Hs) MAP2Ks. Those residues that are phosphorylated by the cognate MAP3Ks are highlighted in red. Numbers refer to the positions in Pbs2. Sc, *Saccharomyces cerevisiae*; Hs, *Homo sapiens*.

B, C Detection of Pbs2 S514 phosphorylation by the Phos-tag band-shift assay. The yeast strain KT003 (*pbs2*Δ) or YM105 (*hog1*Δ *pbs2*Δ) was transformed with YCplac22I'-PBS2-HA (a single-copy plasmid that encoded C-terminally HA-tagged Pbs2 [Pbs2-HA] expressed from the *PBS2* promotor) or its indicated derivatives and was treated with the indicated concentrations of NaCl for 5 min. Cell extracts were immunoprecipitated using anti-HA antibody 3F10 and were subjected to Phos-tag SDS–PAGE. Pbs2-HA was detected by immunoblotting using anti-HA antibody F-7. Positions of phosphorylated and unphosphorylated Pbs2-HA (Pbs2-HA-P and Pbs2-HA-OH, respectively) are indicated.

D, E Detection of Pbs2 T518 phosphorylation by anti-P-T518 immunoblotting. Cell extracts were prepared as in (B) and (C) and were analyzed by standard SDS–PAGE, and immunoblotted for phosphorylated T518 using the anti-P-T518 antibody.

F Mass spectrometric (MS) analysis of Pbs2 phosphorylation. The SHO1 branch-only strain TM280 (*ssk2/22*Δ *pbs2*Δ) or the SNL1 branch-only strain KT005 (*ste11*Δ *pbs2*Δ) was each transformed with pRS414-FLAG-Pbs2. These cells were either unstimulated (0 M NaCl) or stimulated with 0.6 M NaCl for 7 min, and affinity-purified FLAG-Pbs2 was subjected to MS analyses. Numbers of detected peaks that corresponded to the tryptic peptides containing a Pbs2 activating phosphorylation site, either phosphorylated or unphosphorylated, are tabulated.

Source data are available online for this figure.

Figure EV4. Structural basis of the osmotic enhancement of Hog1 phosphorylation by Pbs2.

A Alignment of the amino acid sequences around the DFG motif of various MAP kinases. The positions of N149 and D162 in Hog1, the β7 and β8 strands, the DFG motif, the activation loop, and activating phosphorylation sites (the TXY motif) are indicated. The sequences of the mouse p38α and yeast Hog1 are highly conserved in this segment (23 residues out of 33 are identical, and the other residues show mostly conservative changes). Sc, *Saccharomyces cerevisiae*; Mm, *Mus musculus* (mouse).

B The 3D structure of the mouse p38α MAPK (left), and an annotated enlargement of the relevant segment (residues 151–183; right). The corresponding amino acid sequence is shown in (A). Side chains of N155, D168, and activating phosphorylation sites T180 and Y182 are also shown. N155 and D168 correspond to, respectively, the yeast Hog1 residues N149 and D162, whose mutations created the constitutively enhanced phenotype. The coordinate data were from PDB (ID 5UOJ) (Wang *et al*, 1997) and were visualized using the MOLMOL program (Koradi *et al*, 1996).

C The 3D structure of the mouse p38α MAPK showing the spatial relationship between the L16 domain and the DGF motif. Four side views of the mouse p38α are shown, each of which was rotated 90° from the previous one around the vertical axis. Following segments are highlighted by coloring: the DFG motif (brown), the CD domain (green), and the L16 domain (pink).

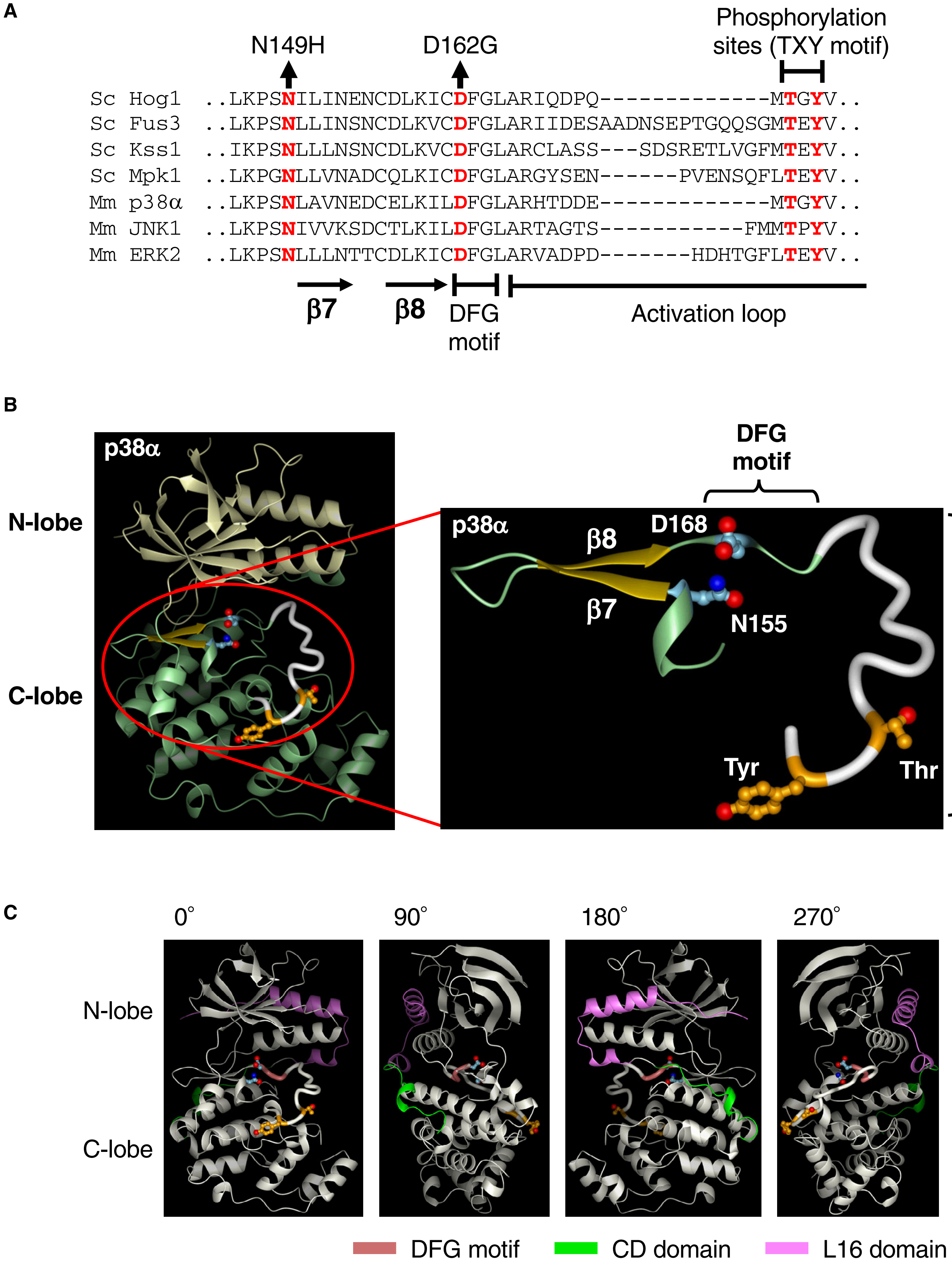

Figure EV4.

 © 2020 The Authors

Figure EV5. Summary and hypothetical models.

A A hypothetical mechanism of the osmotic enhancement of the Pbs2-Hog1 reaction. The first step is an osmotic conversion of Hog1 from a poor substrate of Pbs2 to a better substrate of Pbs2 and is mimicked by Hog1 N149H/D162G mutation. The second step is phosphorylation of the Hog1 activation loop by Pbs2. Two ovals represent the N- and C-lobes of Hog1 kinase domain, and the red curve represents the activation loop.

B A schematic comparison of the HOG pathway and the pheromone pathway. In both pathways, the specific stimulus acts twice: first at the cell surface receptor/sensor, and second at the step of MAP2K-MAPK reaction.

C–E Three modes of the Hog1 activation time courses are explained by negative and positive feedback regulations. In the left panels, the dose–response curves are taken from Fig 1I (error bars are omitted). Three colored arrows indicate the applied external NaCl osmolarities and symbolize the gradual decrease of effective osmostress with time. In the right panels, time courses at three NaCl concentrations are taken from Fig 9B–D, and shown by the same colors used in the corresponding left panels.

F Hypothetical model of the evolutionary development of the three-kinase MAPK cascades.

© 2020 The Authors

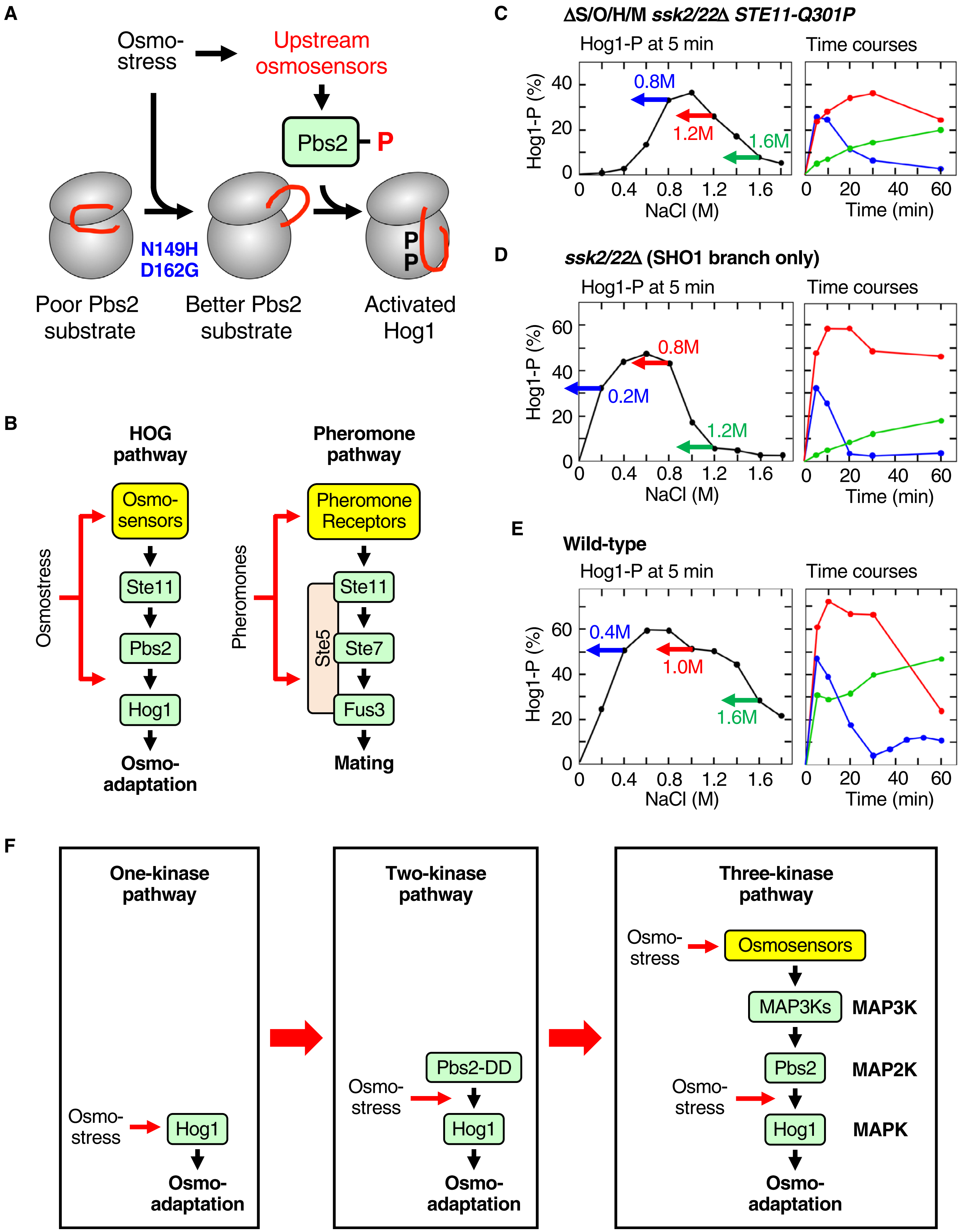

Figure EV5.

© 2020 The Authors
