# Supplementary figures and images for: Osmostress enhances activating phosphorylation of Hog1 MAP kinase by mono‐phosphorylated Pbs2 MAP2K
Source: EMBO J. 2020 Feb 3;39(5):e103444. doi: 10.15252/embj.2019103444 (PMC7049814; doi:10.15252/embj.2019103444)

Source Data Figure EV1

EV1C

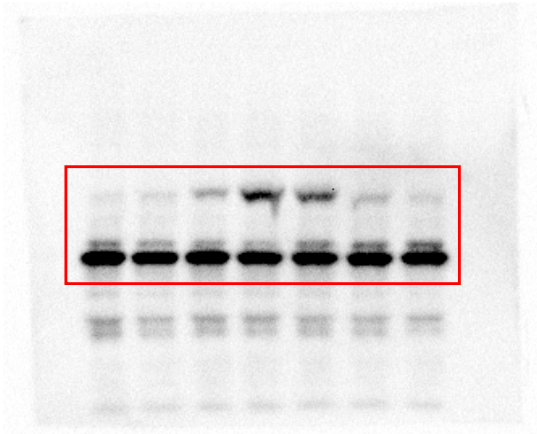

160301-osmo4QP-sorb (AN)

Supplement: Supplementary file 3 — Source Data for Expanded View [file EMBJ-39-e103444-s012.zip › EMBOJ_103444_SourceData_FigEV1.pdf]

Source Data Figure EV3

EV3B

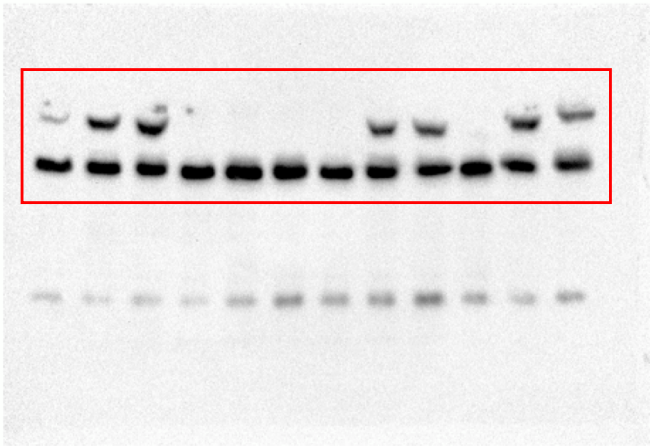

170831 phos-tag HA

EV3C

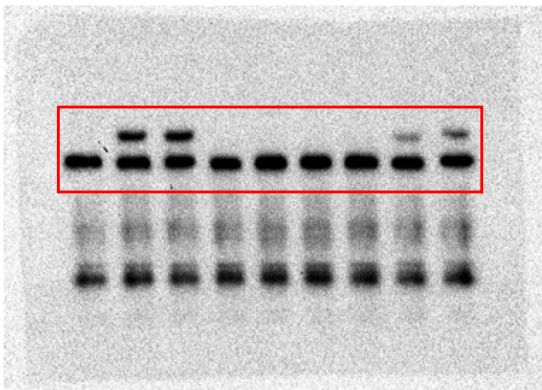

190307 A1-3 0.4,1.0M NaCl Phos-tag HA(F-7)

EV3D

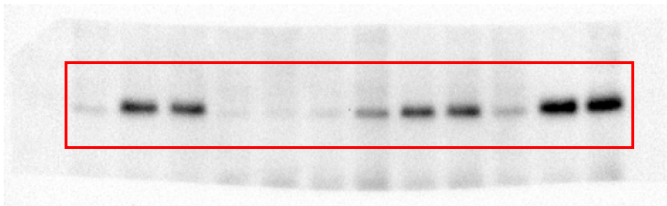

190612 T1,4,5,a1 p-T518 Pbs2 (scrum)

EV3E

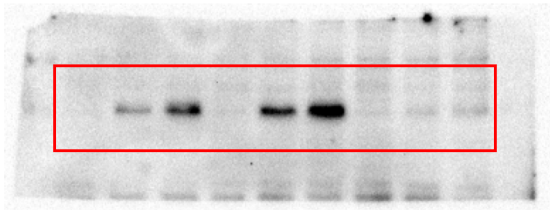

190620 T1-3 p-T518(scrum)

Supplement: Supplementary file 3 — Source Data for Expanded View [file EMBJ-39-e103444-s012.zip › EMBOJ_103444_SourceData_FigEV3.pdf]

Source Data Figure 1

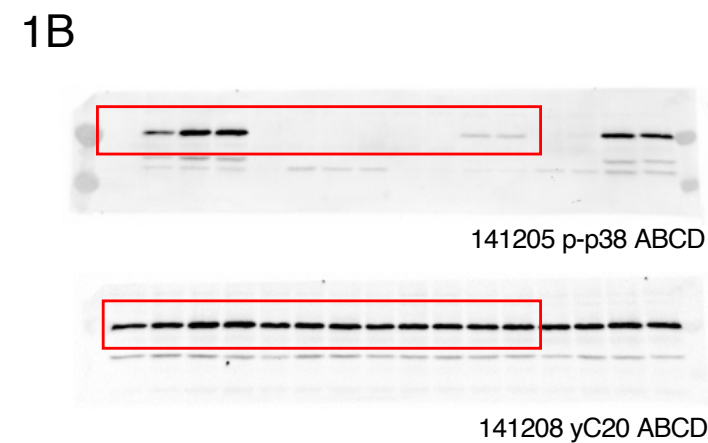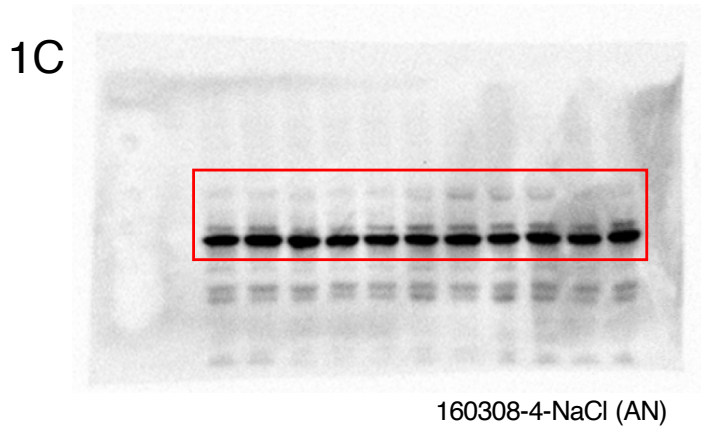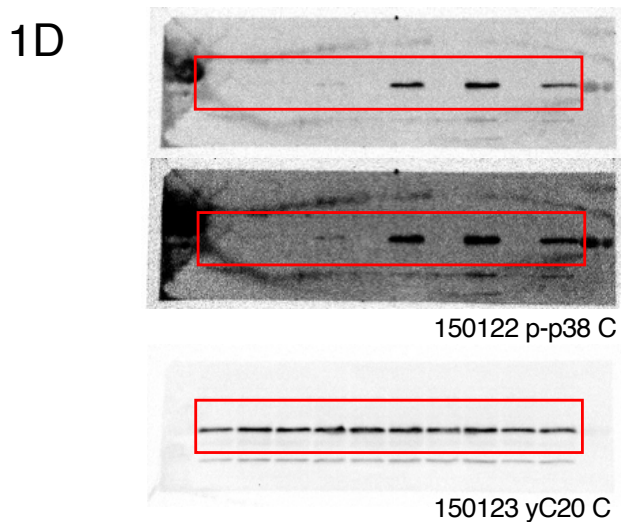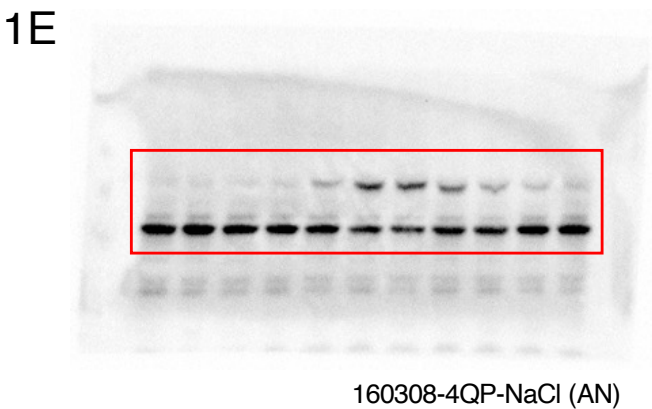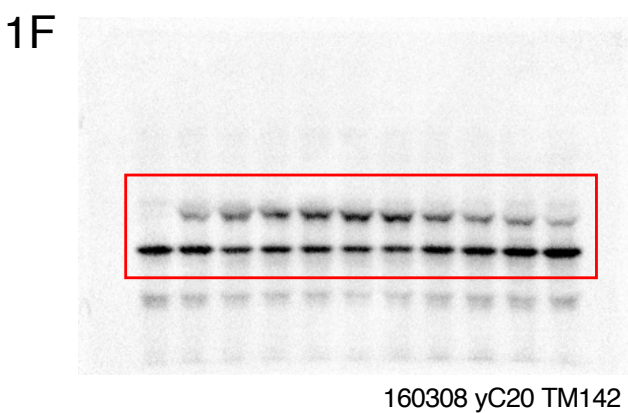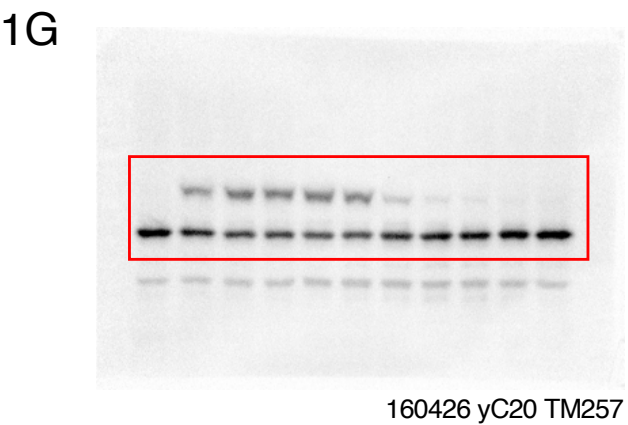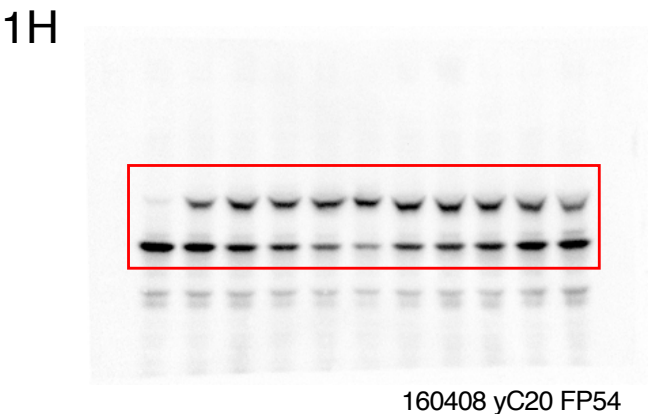

Supplement: Supplementary file 5 — Source Data for Figure 1 [file EMBJ-39-e103444-s003.pdf]

Source Data Figure 2

2A

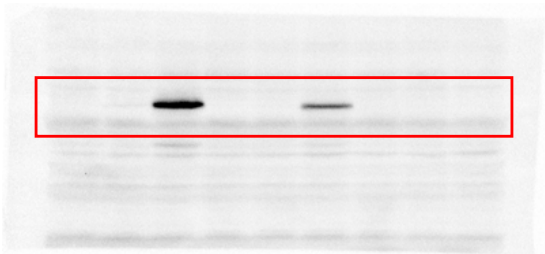

160318 p-p38 a

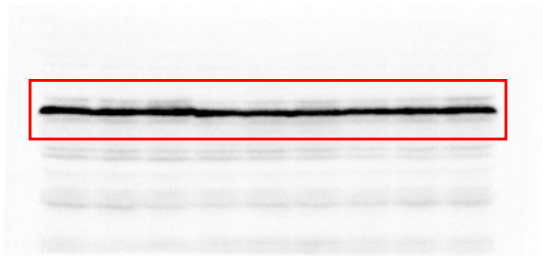

160318 yC20 a

2B

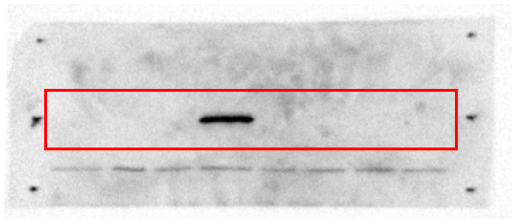

151215 p-p38 g

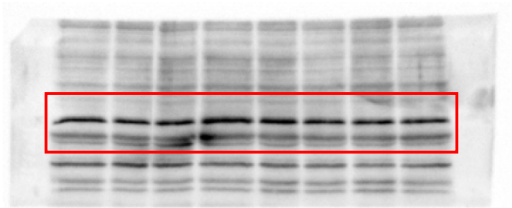

151215 yC20 g

2C

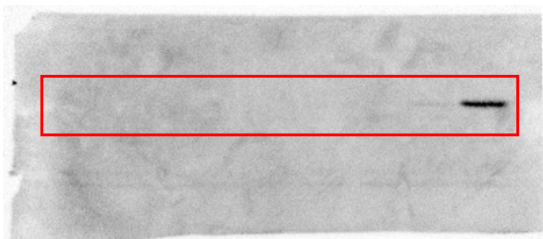

160120 p-p38 k

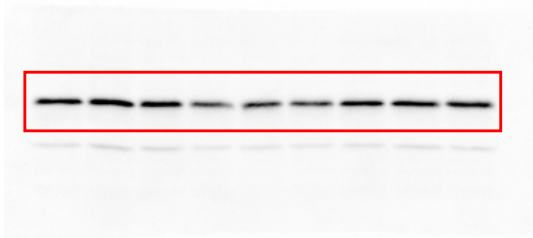

160120 yC20 k

2D

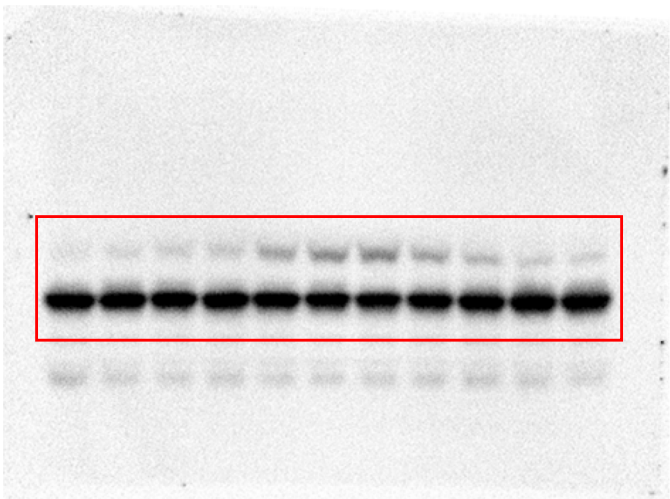

180216 NaCl Phos-tag(yC20)

Supplement: Supplementary file 6 — Source Data for Figure 2 [file EMBJ-39-e103444-s004.pdf]

Source Data Figure 3

3A

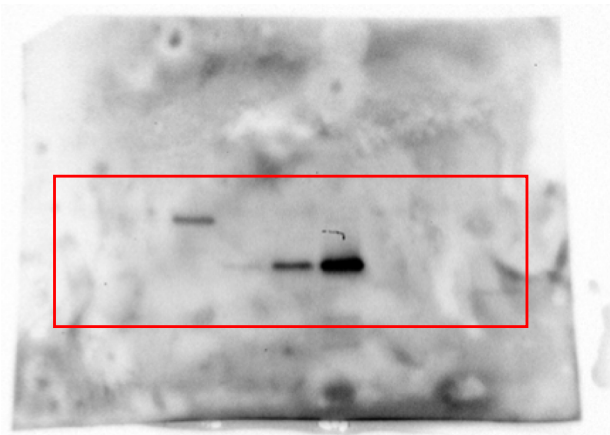

190411 E1-3 IP p-p38(9211S)

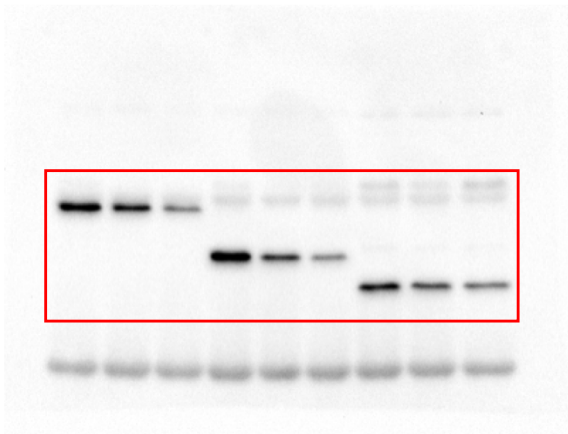

190411 E1-3 IP reblot FLAG(M2)

3D

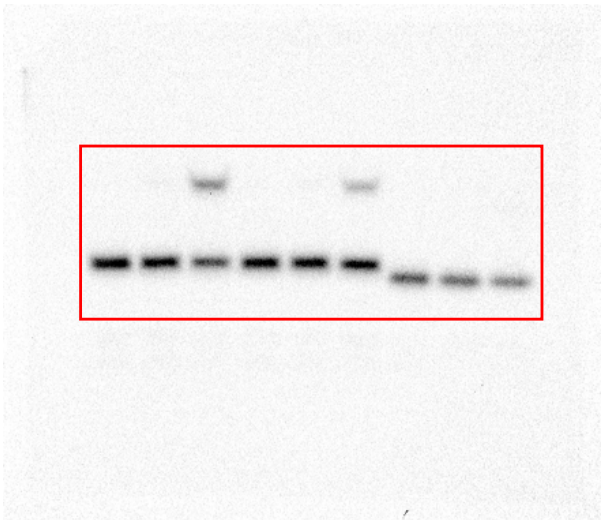

181228 n Phos-tag Hog1(yC20)

3F

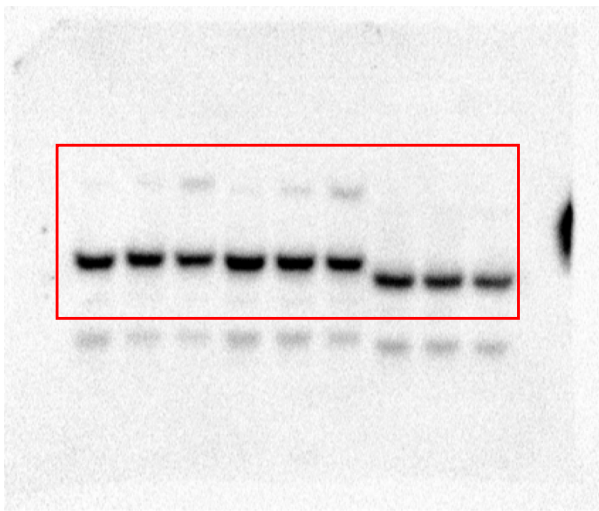

190409 F4-6,1 Phos-tag Hog1(yC20)

Supplement: Supplementary file 7 — Source Data for Figure 3 [file EMBJ-39-e103444-s005.pdf]

Source Data Figure 4

4A

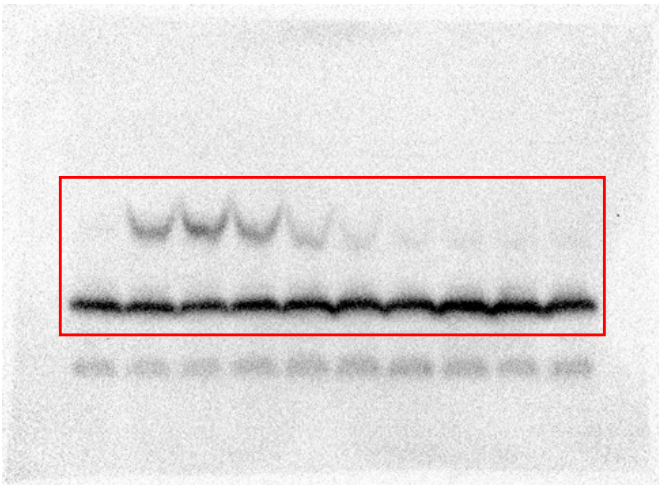

191108 A1-BK Phos-Tag Hog1(yC20)

4C

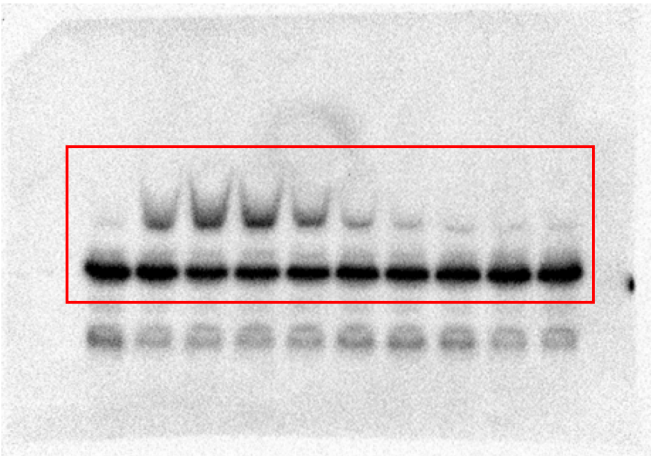

190131 l3 Phos-tag Hog1(yC20)

4E

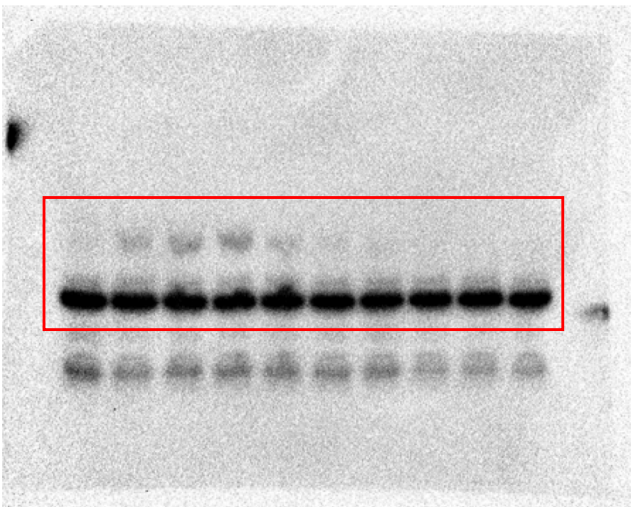

190131 k3 Phos-tag Hog1(yC20)

Supplement: Supplementary file 8 — Source Data for Figure 4 [file EMBJ-39-e103444-s006.pdf]

Source Data Figure 7

7A

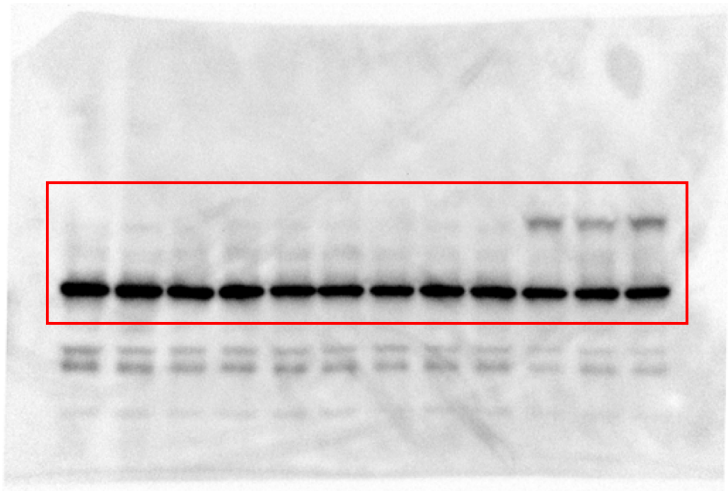

170412 F yC20

Supplement: Supplementary file 11 — Source Data for Figure 7 [file EMBJ-39-e103444-s009.pdf]

Source Data Figure 8

8A

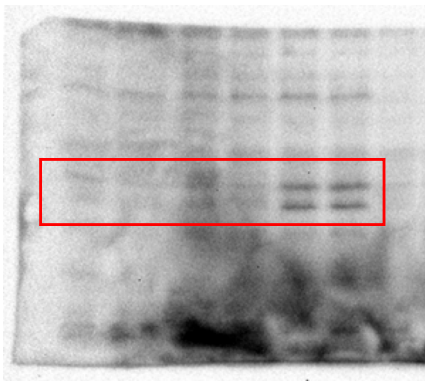

190712 KT299 p-p44 42

8B

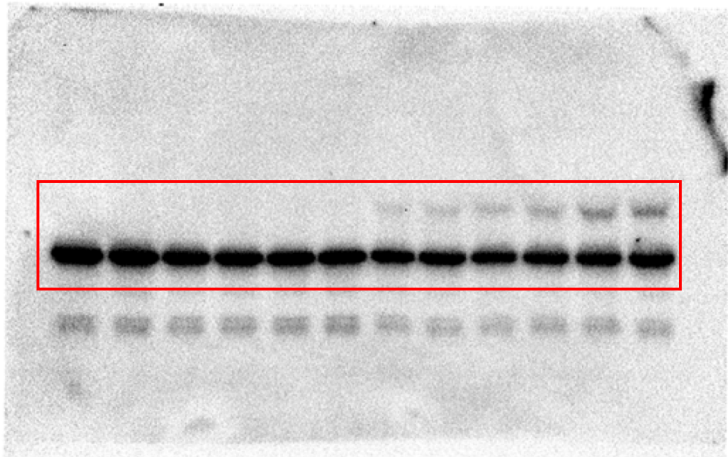

190710 KT299 Phos-tag Hog1(yC20)

Supplement: Supplementary file 12 — Source Data for Figure 8 [file EMBJ-39-e103444-s010.pdf]

Source Data Figure 9

9A

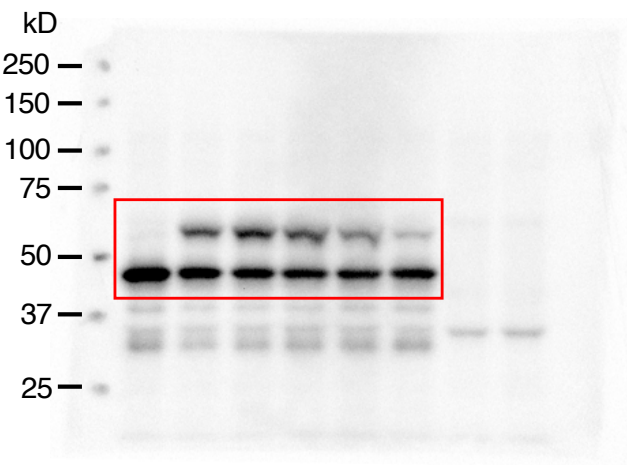

151230 yC20 KY603-3 1M

Supplement: Supplementary file 13 — Source Data for Figure 9 [file EMBJ-39-e103444-s011.pdf]
