# Supplementary material for: Osmostress enhances activating phosphorylation of Hog1 MAP kinase by mono‐phosphorylated Pbs2 MAP2K
Source: EMBO J. 2020 Feb 3;39(5):e103444. doi: 10.15252/embj.2019103444 (PMC7049814; doi:10.15252/embj.2019103444)
Supplement: Supplementary file 9 — Source Data for Figure 5 [file EMBJ-39-e103444-s007.pdf]

Source Data Figure 5

5A

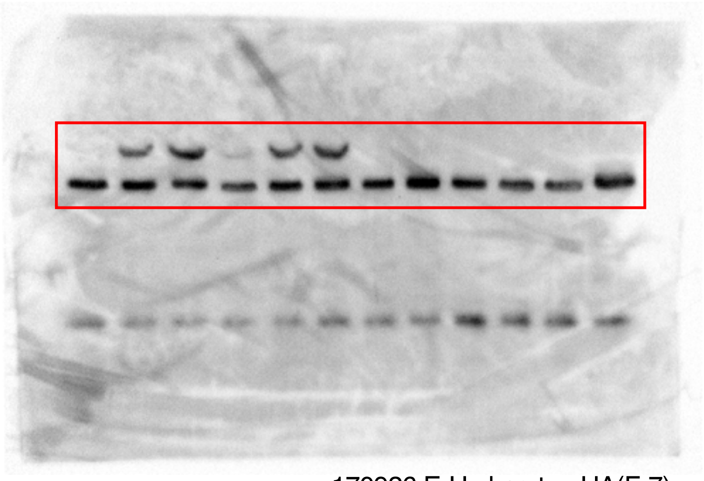

170926 E-H phos-tag HA(F-7)

5B

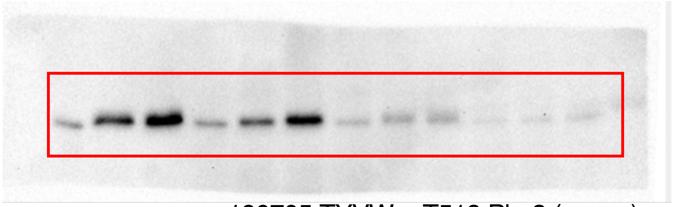

190705 TYVW o-T518 Pbs2 (scrum)

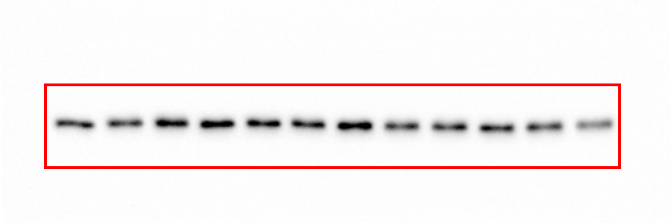

190705 TUVW HA(F-7)

5C

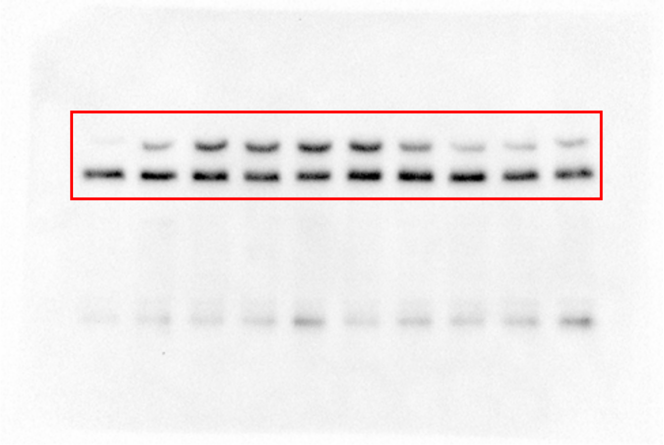

190305 B Phos-tag HA(F-7)

5D

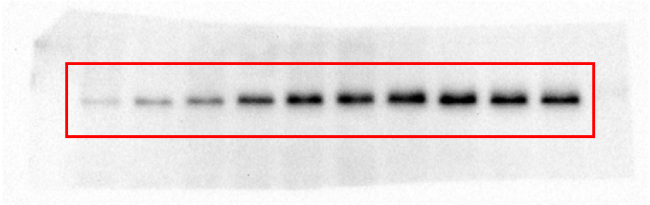

190704 U1 p-T518 Pbs2(scrum)

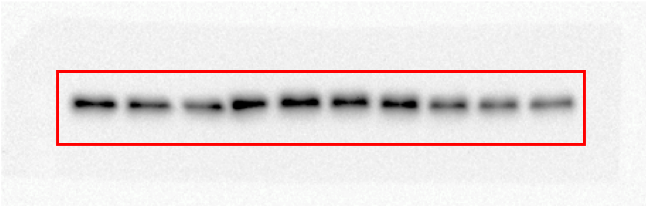

190704 U1 HA(F-7)

5E

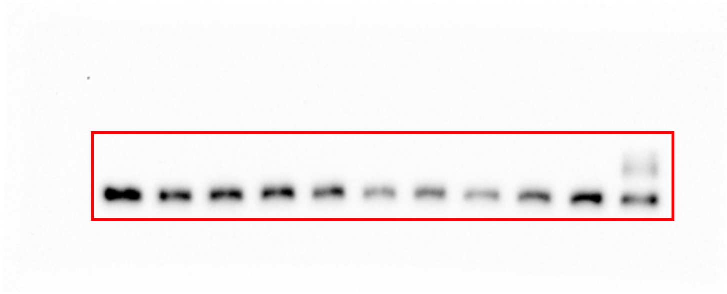

190627 V1 Phos-tag HA(F-7)

5F

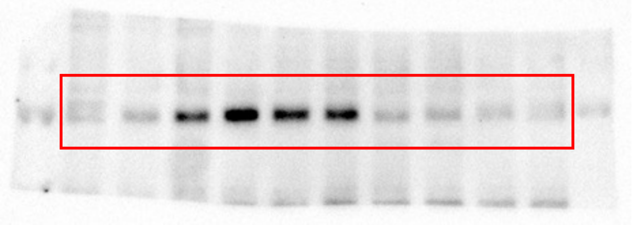

190628 V1 p-T518 Pbs2(scrum)

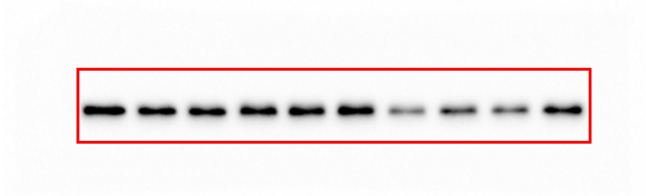

190628 V1 HA(F-7)

5I

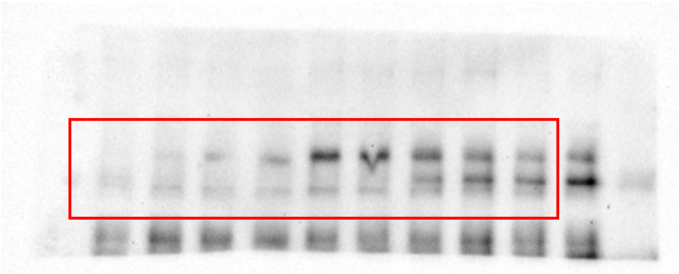

190703 U1 Phos-tag P-T518(scrum)

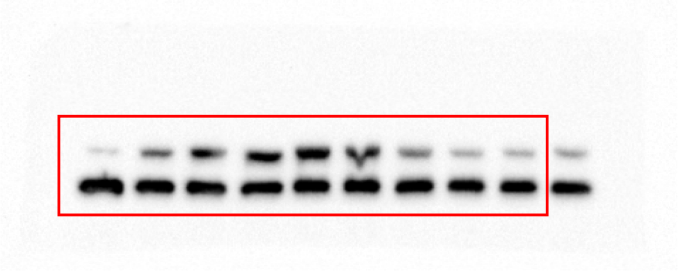

190703 U1 reblot HA(F-7)

5J

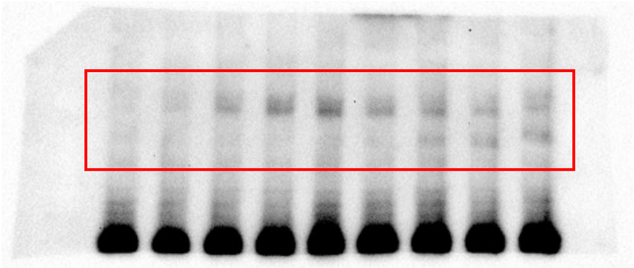

191115 B Phos-tag P-T518(scrum)

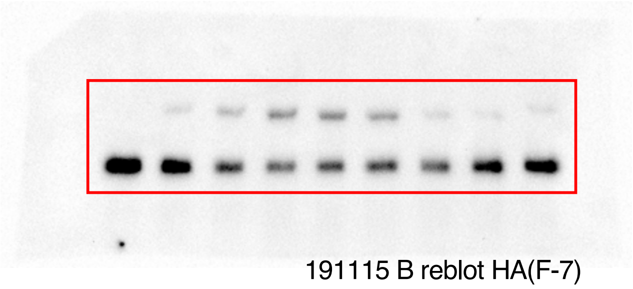

191115 B reblot HA(F-7)
