# Supplementary material for: Osmostress enhances activating phosphorylation of Hog1 MAP kinase by mono‐phosphorylated Pbs2 MAP2K
Source: EMBO J. 2020 Feb 3;39(5):e103444. doi: 10.15252/embj.2019103444 (PMC7049814; doi:10.15252/embj.2019103444)
Supplement: Supplementary file 10 — Source Data for Figure 6 [file EMBJ-39-e103444-s008.pdf]

Source Data Figure 6

6B

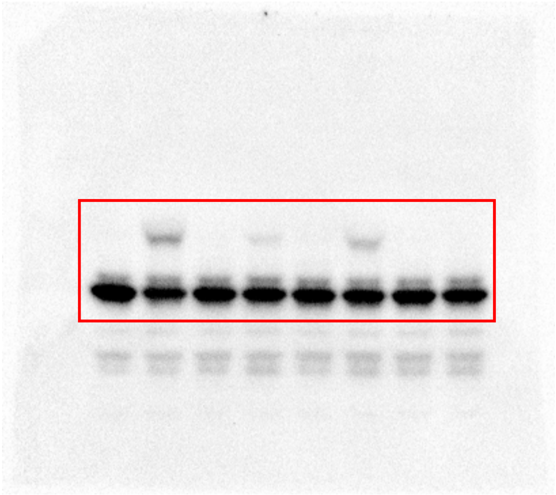

180705 B1-4 0.6M NaCl Ly Phos-tag(yC20) re

6C

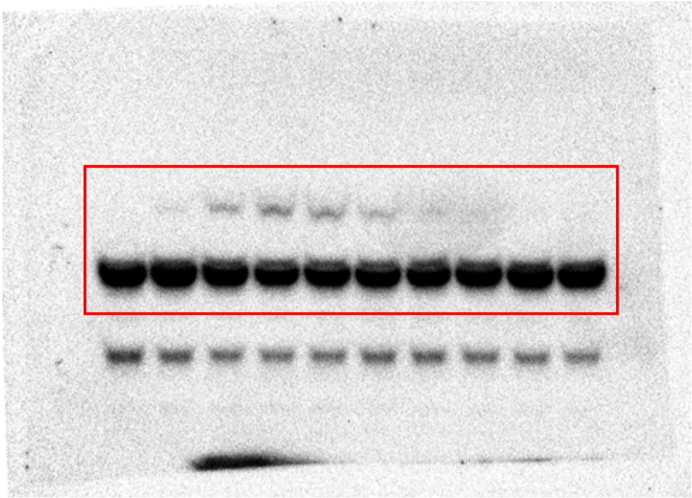

190530 Y5-2 Phos-tag Hog1(yC20)

6D

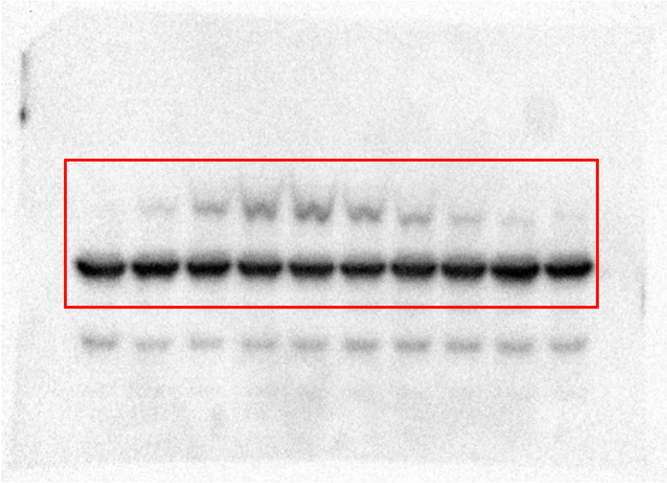

190308 D1 Phos-tag Hog1(yC20)

6E

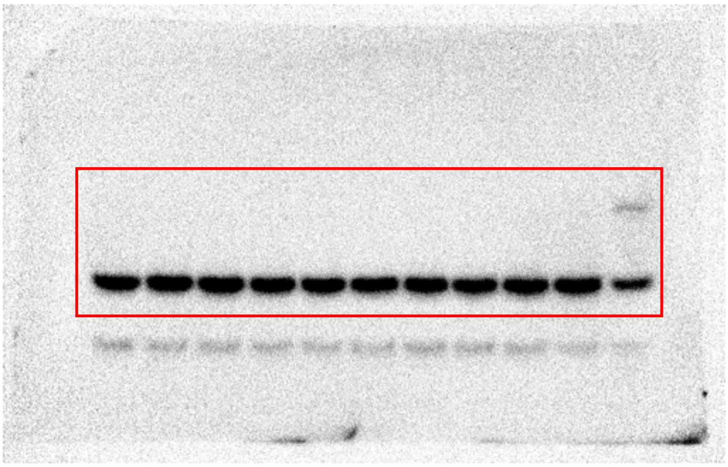

190611 Y6-2 Phos-tag Hog1(yC20)

6F

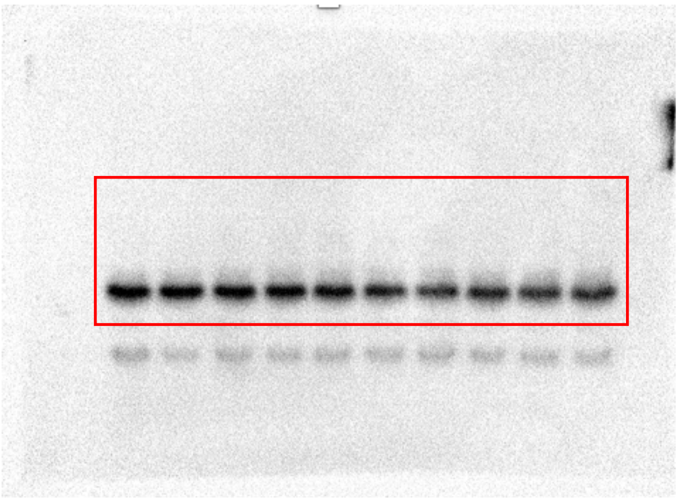

190308 D2 Phos-tag Hog1(yC20)
